# Correction: Exploiting the inherent promiscuity of the acyl transferase of the stambomycin polyketide synthase for the mutasynthesis of analogues

**DOI:** 10.1039/d5sc90046h

**Published:** 2025-03-07

**Authors:** Li Su, Yaouba Souaibou, Laurence Hôtel, Christophe Jacob, Peter Grün, Yan-Ni Shi, Alicia Chateau, Sophie Pinel, Helge B. Bode, Bertrand Aigle, Kira J. Weissman

**Affiliations:** a Université de Lorraine, CNRS, IMoPA F-54000 Nancy France kira.weissman@univ-lorraine.fr; b Université de Lorraine, INRAE, DynAMic F-54000 Nancy France bertrand.aigle@univ-lorraine.fr; c Max-Planck-Institute for Terrestrial Microbiology, Department of Natural Products in Organismic Interactions 35043 Marburg Germany; d IPHC, UMR 7178, CNRS, Université de Strasbourg, Equipe de Chimie Analytique des Molécules Bioactives et Pharmacognosie Illkirch France; e Molecular Biotechnology, Department of Biosciences, Goethe University Frankfurt Frankfurt am Main Germany; f Université de Lorraine, CNRS, CRAN F-54000 Nancy France; g Chemical Biology, Department of Chemistry, Philipps University of Marburg 35043 Marburg Germany; h Senckenberg Gesellschaft für Naturforschung 60325 Frankfurt am Main Germany; i Center for Synthetic Microbiology (SYNMIKRO), University of Marburg 35043 Marburg Germany

## Abstract

Correction for ‘Exploiting the inherent promiscuity of the acyl transferase of the stambomycin polyketide synthase for the mutasynthesis of analogues’ by Li Su *et al.*, *Chem. Sci.*, 2025, https://doi.org/10.1039/d4sc06976e.

The authors hereby amend the Acknowledgements section of their published article to thank Dr Benjamin Chagot for NMR analysis of certain fed substrates.

The Royal Society of Chemistry apologises for these errors and any consequent inconvenience to authors and readers.

